# High-Rate One-Dimensional α-MnO_2_ Anode for Lithium-Ion Batteries: Impact of Polymorphic and Crystallographic Features on Lithium Storage

**DOI:** 10.3390/nano13202808

**Published:** 2023-10-23

**Authors:** Hye-min Kim, Byung-chul Cha, Dae-wook Kim

**Affiliations:** 1 Department of Materials Chemistry, Shinshu University, 4-17-1, Wakasato, Nagano 3808553, Japan; hmkim545@gmail.com; 2 Advanced Manufacturing Process R&D Group, Ulsan Division, Korea Institute of Industrial Technology (KITECH), 55, Jongga-ro, Jung-gu, Ulsan 44313, Republic of Korea

**Keywords:** Li-ion batteries, α-MnO_2_, anode, Li-ion transport, aspect ratio

## Abstract

Manganese dioxide (MnO_2_) exists in a variety of polymorphs and crystallographic structures. The electrochemical performance of Li storage can vary depending on the polymorph and the morphology. In this study, we present a new approach to fabricate polymorph- and aspect-ratio-controlled α-MnO_2_ nanorods. First, δ-MnO_2_ nanoparticles were synthesized using a solution plasma process assisted by three types of sugars (sucrose, glucose, and fructose) as reducing promoters; this revealed different morphologies depending on the nucleation rate and reaction time from the molecular structure of the sugars. Based on the morphology of δ-MnO_2_, the polymorphic-transformed three types of α-MnO_2_ nanorods showed different aspect ratios (*c/a*), which highly affected the transport of Li ions. Among them, a relatively small aspect ratio (*c/a* = 5.1) and wide width of α-MnO_2_-S nanorods (sucrose-assisted) induced facile Li-ion transport in the interior of the particles through an increased Li-ion pathway. Consequently, α-MnO_2_-S exhibited superior battery performance with a high-rate capability of 673 mAh g^−1^ at 2 A g^−1^, and it delivered a high reversible capacity of 1169 mAh g^−1^ at 0.5 A g^−1^ after 200 cycles. Our findings demonstrated that polymorphs and crystallographic properties are crucial factors in the electrode design of high-performance Li-ion batteries.

## 1. Introduction

Manganese oxides with various valence states (+II, +III, and +IV) have attracted considerable interest because of the variety of their crystal structures and unique properties [1,2]. They have a wide range of potential applications in energy conversion and storage, such as lithium batteries, zinc-air batteries, capacitors, fuel cells, and solar cells, because of their low cost, environmental friendliness, and electrochemical properties in multiple oxidation states [3,4,5,6,7,8].

Research on manganese oxides as anode materials in Li-ion battery (LIBs) systems has increasingly emerged because exploding electrical vehicles (EVs) demand advanced LIB performance. Higher energy and power densities compared to those of the current LIBs system are required to increase the driving distance and achieve quick charging [9,10]. In LIBs, although commercialized graphite anodes exhibit high stability at low operating voltages, their small theoretical capacity (372 mAh g^−1^) is insufficient to achieve high energy density [11]. Transition metal oxides (TMOs) are promising alternatives as conversion anode materials because of their high specific capacity and low cost [12,13]. Among them, manganese oxides are outstanding because of their high theoretical capacity and low operating voltage with low-voltage hysteresis compared to other TMOs such as iron, nickel, and cobalt oxides [14,15].

The battery performance of manganese oxides is directly attributed to their oxidation state, morphology, and nanostructure. Among the various manganese oxides, MnO_2_ has the highest theoretical capacity of 1232 mAh g^−1^ because of the multiple steps of reversible conversion reaction from the Mn^4+^ ↔ Mn^3+^ ↔ Mn^2+^ ↔ Mn^0^ state during lithiation/delithiation. However, a higher capacity with multiple conversion steps inevitably involves large volumetric expansion during cycling, which can degrade the performance [15,16,17]. Structural optimization, including morphological control and nanostructuring, is necessary to overcome this problem. Numerous studies have focused on controlling nanostructured MnO_2,_ such as spheres, wires, sheets, and rods [18,19]. Further, MnO_2_ exists in several polymorphic forms based on the MnO_6_ octahedral interlinked configurations in different manners [20]. These forms include a one-dimensional tunnel type (α-MnO_2_, tetragonal with *I*4*/m* space group; β-MnO_2_, tetragonal with *P*4_2_*/mnm* space group; γ-MnO_2_, orthorhombic with *Pnma* space group), two- dimensional layered type (δ-MnO_2_, monoclinic with *C*2/m space group), and a three-dimensional spinel type (λ-MnO_2_, cubic with space group *Fd*-3*m*), which can affect to electrochemical properties [21,22]. Theoretically, 1 × ∞ layered structure of δ-MnO_2_ and the 2 × 2 tunnel structure of α-MnO_2_ provide sufficient Li-ion transport pathways; further, the large layer interspacing of δ-MnO_2_ is favorable for ion diffusion [23,24]. Hence, appropriate polymorphs with morphology-controlled nanostructures are key to overcoming performance limitations.

Hydrothermal, sol-gel, and solid-state reactions are used to synthesize α-MnO_2_ with various crystal structures and morphologies. However, these methods require the use of toxic reduction or oxidation agents such as HCl, HNO_3_, and H_2_SO_4_ with long processing times. Considering the recent environmental issues, toxic chemical agents are required to replace the green chemicals in natural extracts. Similarly, there has been an increase in the use of simple and energy-saving synthesis routes in materials science and chemistry [25,26]. Therefore, in this study, we introduce a solution-plasma process (SPP) as an innovative synthetic route to nanostructured δ-MnO_2_. The SPP, which creates a nonequilibrium plasma at the liquid interface, generates numerous energetic electrons and reactive species that promote rapid reduction or oxidation reactions in aqueous solutions [27,28]. This novel chemical reaction is widely used in the synthesis of advanced nanomaterials, including carbon, metal oxides, and inorganic-organic hybrids. In our previous study, a potassium permanganate solution was effectively reduced to colloidal MnO_2_ during plasma discharge [29]. Three sugars (sucrose, glucose, and fructose) were used as reducing promoters to control the processing time and nucleation rate. The three types of δ-MnO_2_ revealed differences in their nanostructures because of the sugar types with inherent molecular structures. The crystal shape and size were controlled by post-calcination to clarify the effect of the polymorphic structural features on the electrochemical properties. We report shape effects on battery performance through systematic investigations between nanoparticles and nanorods of α-MnO_2_. Further, determining the effect of the aspect ratio on α-MnO_2_ nanorods provides information on how crystallographic features impact the Li-ion transport behavior. We believe that a shape and size-controlled α-MnO_2_ nanostructure can provide experimental evidence for conversion anode materials design to achieve high-energy-density LIBs.

## 2. Materials and Methods

### 2.1. Preparation of δ-MnO_2_ and One-Dimensional α-MnO_2_

**Figure 1:**
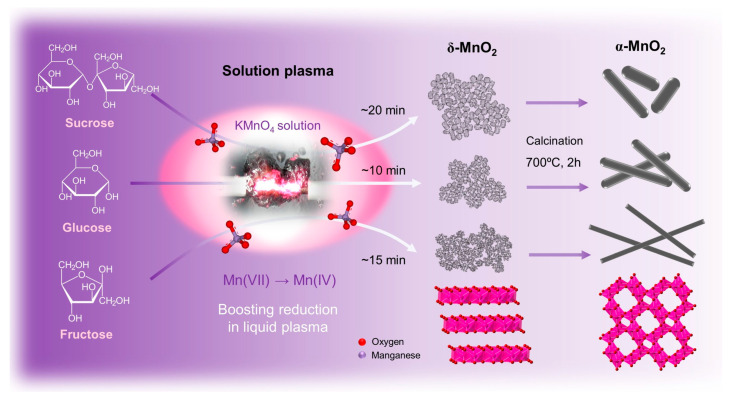
Schematic of the solution plasma-sugar assisted synthesis of δ-MnO_2_ and post-calcination for α-MnO_2_.

Three types of birnessite δ-MnO_2_ were prepared by solution plasma and a sugar-assisted reduction reaction. The synthesis procedure is illustrated in Figure 1. First, 0.08 M of potassium permanganate (KMnO_4_, >99.3%, DAEJUNG Chemicals & Metals Co., Ltd., Shiheung-city, Korea) is dissolved in 80 mL of distilled water with vigorous stirring. Then, 0.01 M of each sucrose (C_12_H_22_O_11_, >99.9%), glucose (C_6_H_12_O_6_, >99.9%), and fructose (C_6_H_12_O_6_, >99.9%) purchased from DAEJUNG Chemicals & Metals Co., Ltd., was dissolved in 20 mL of distilled water. After thorough mixing, 80 mL of KMnO_4_ and 20 mL of each sugar solution (sucrose, glucose, and fructose) were transferred to a 100 mL volume of the reactor. The experimental setup for SPP is shown in Figure S1. A bipolar pulse power supply (MPP04, Kurita Manufacturing Co., Ltd., Kyoto, Japan) was connected to a pair of Φ1 mm tungsten metal electrodes to generate plasma in the solution. The frequency (20 kHz), pulse width (2.5 μs), and electrode gap (1 mm) were adjusted, and plasma was generated in each mixed precursor. The purple KMnO_4_ aqueous solution completely turned into a solid particle and transparent solution within ~20 min. The resulting products were gathered through vacuum filtration using a membrane filter, rinsed with distilled water, and dried in a vacuum oven under 100 °C. As-obtained products were donated to δ-MnO_2_-S (using sucrose), δ-MnO_2_-G (using glucose), and δ-MnO_2_-F (using fructose), respectively. As prepared, three types of δ-MnO_2_ were conducted post-calcination at 700 °C for 2 h under atmospheric conditions to obtain the α-MnO_2_ phase and one-dimensional structure. The obtained product was designated by α-MnO_2_-S, α-MnO_2_-G, and α-MnO_2_-F, respectively.

### 2.2. Material Characterization

X-ray diffraction (XRD) analysis was performed using a Rigaku Ultima IV system with Cu Kα radiation (λ = 1.5418 α), operating voltage of 45 kV, and current of 200 mA in steps of 0.02°. The morphology was investigated using field-emission scanning electron microscopy (FE-SEM, SU8020, Hitachi, Tokyo, Japan) at an acceleration voltage of 5 kV and transmission electron microscopy (TEM, JEL-2500SE, JEOL, Tokyo, Japan) at an acceleration voltage of 200 kV. The Brunauer–Emmett–Teller (BET) surface area and Barrett–Joyner–Halenda (BJH) pore size distributions were analyzed using an ASAP 2020 (Micromeritics). X-ray photoelectron spectroscopy (XPS) measurement was carried out through the NEXSA system. The particle size distributions of length and width were evaluated using ImageJ software (version 1.53t).

### 2.3. Electrochemical Measurements

An electrode slurry was prepared by mixing (AR-100, Thinky) active materials (AM, δ-MnO_2_-S, -G, -F, and α-MnO_2_- S, -G, and -F), conducting agent (acetylene black), and binder (polyacrylic acid) in a weight ratio of 8:1:1 (wt%). The as-prepared slurry was coated on a Cu foil using a doctor’s blade and adjusted to ~1.5 mg cm^−2^ of the loading amount. The 1 M LiPF_6_ in a mixture of ethylene carbonate and dimethyl carbonate (3:7 vol. % (Enchem Co., Ltd., Cheonan-si, Republic of Korea) was used as the electrolyte. The 2032-type coin-half-cell was utilized for the electrochemical tests. The coin half-cell consisted of lithium metal as the counter electrode, a microporous membrane as the separator (Celgard 2500), and the electrolyte. All coin cells were assembled in an argon-purged glove box (GBI Co., Ltd., New York, NY, USA). Galvanostatic charge-discharge, rate, and cycle tests were performed using a battery tester WBCS3000Le (WONATECH). All electrochemical performances were measured in the potential range of 0.01–3.0 V (vs. Li/Li^+^). The cyclic voltammetry (CV) and electrochemical impedance spectroscopy (EIS) were performed using a Zive MP2A potentiostat (WONATECH). The CV curve was obtained at a scan rate of 0.1 mV, and EIS was measured in the frequency range of 200 kHz to 0.01 Hz. The rate capability was investigated at different current densities of 0.1, 0.2, 0.5, 1, 2, and 0.5 A g^−1^ for five cycles under 25 °C. Cycle tests were performed with a current density of 0.5 A g^−1^ for 200 cycles at 25 °C.

## 3. Results

Nanostructured MnO_2_ was synthesized using different sugars (glucose, fructose, and sucrose) as reducing promoters to improve the reaction yield and reduce the processing time, as shown in Figure 1. Different types of sugars containing different functional groups, such as aldehydes (–CHO) in glucose and ketones (–C=O) in fructose, can play essential roles as reducing agents, which results in products with different properties [30,31]. Although sucrose is a nonreducing sugar held between glucose and fructose by glycosidic linkages, sucrose can be decomposed into two types of reducing sugars, glucose and fructose, by applying plasma discharge [32]. Therefore, sucrose acts as a reducing agent in the plasma. We defined the reaction time as the time when the purple solution completely changed to a transparent solution and brown particles. The reaction times of approximately 10 (glucose), 15 (fructose), and 20 min (sucrose) were obtained. These results suggest that the reduction rate depends on the sugar type (i.e., functional groups). It is because glucose is a strong reducing agent due to the presence of hydrogen-containing aldehyde groups that a fast reduction reaction occurs. However, fructose, which contains the ketone group, must undergo keto−enol tautomerism in order to act as reducing sugars, and thus, the reaction time for MnO_2_ formation is longer than glucose. Therefore, these inherent properties of each sugar can affect the particle size and shape [33,34]. In addition, to confirm the effect of the simultaneous application of sugar and SPP, the reduction time (MnO_4_^−^ to MnO_2_) was measured when only SPP or sugar was used. Our sugar-assisted SPP was considerably faster than that of each (SPP: >60 min and chemical precipitation with sugar: >40 h), indicating the synergistic effect of SPP and sugar in accelerating the reduction reaction. Subsequently, crystal shape and size were controlled by post-calcination (700 °C, 2 h) to verify the effect of structural features with a polymorphic form on electrochemical properties. The selection of a suitable reducing promoter is a key factor because the particle size and shape are strongly dependent on its intrinsic properties.

**Figure 2:**
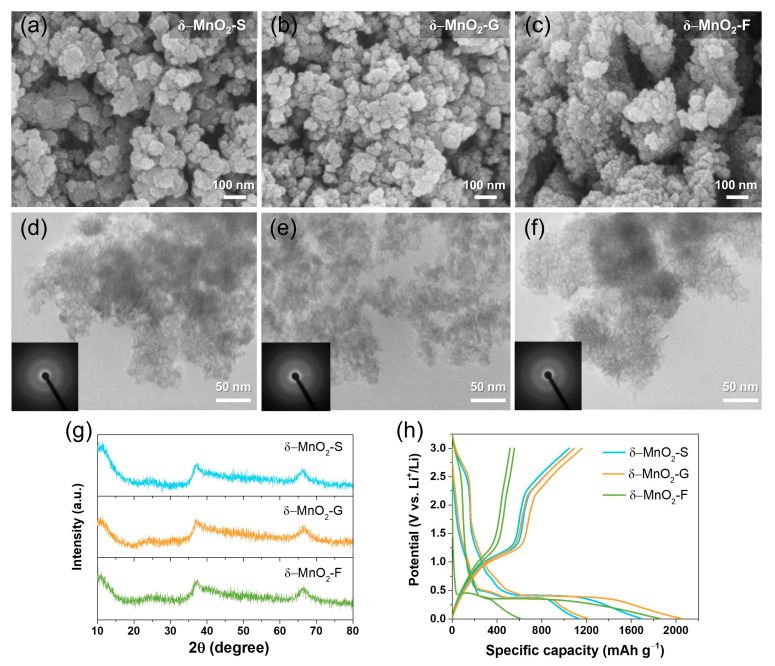
FE-SEM and TEM images (inset SAED pattern) of (**a**,**d**) δ-MnO_2_-S, (**b**,**e**) δ-MnO_2_-G, and (**c**,**f**) δ-MnO_2_-F. (**g**) XRD patterns, (**h**) galvanostatic charge–discharge curves for the first and second cycles of δ-MnO_2_ electrodes.

First, we discuss the SPP-sugar-assisted synthesis products. XRD and electron microscopy were performed to verify the structure and morphology. Figure 2g shows that the XRD patterns of as-obtained products synthesized by SPP with sucrose, glucose, and fructose, respectively, presented birnessite type δ-MnO_2_ (JCPDS 80-1098). In addition, no sharp peaks were observed for any of the samples, indicating that the obtained materials were amorphous. Figure 2a–c shows the FE-SEM images of δ-MnO_2_ particles, which differed slightly depending on the sugar type. The δ-MnO_2_-S shows randomly aggregated nanoparticles comprising irregularly shaped small nanoparticles in the range of 20–200 nm (Figure 2a). For δ-MnO_2_-G, comparatively smaller aggregated particles were observed than δ-MnO_2_-S, which indicates the sub-100 nm size. Finally, δ-MnO_2_-F shows the largest aggregates, which comprise the smallest primary particles of 10–20 nm. The particle size and morphology were more clearly observed in the TEM images (Figure 2d–f), and selected area electron diffraction (SAED) patterns (inset Figure 2d–f) revealed a diffused ring demonstrating an amorphous structure. Differences in particle size and morphology could be related to the inherent reduction of promoters. When sucrose was introduced, it decomposed into glucose and fructose, thereby supporting the reduction of MnO_4_^−^ to MnO_2_. This complicated reaction causes a slow nucleation rate and longer reaction times, which result in a larger particle size distribution compared to that of the others [35,36]. Although fructose showed longer reaction times than glucose, it led to a smaller particle size distribution. We assumed that this result might be associated with their inherent molecular structures. A more open-chain form of fructose induces a faster reaction at the initial stage because fructose exists in a higher fraction of linear structures than glucose, and this leads to a faster nucleation rate in fructose [37]. Owing to the small particle size, δ-MnO_2_-F showed the largest specific surface area (SSA) among all samples, and the order of SSA was as follows: δ-MnO_2_-F > δ-MnO_2_-G > δ-MnO_2_-S (Figure S2, Table S1). All samples presented hierarchical mesoporous structures.

Galvanostatic charge–discharge and rate capability tests were performed to compare the dependence of the electrochemical performances of δ-MnO_2_ on the sugar type. Figure 2h shows initial specific discharge (lithiation)/charge (delithiation) capacity and initial coulombic efficiency (ICE): 1689 and 1090 mAh g^−1^ with 64.5% for δ-MnO_2_-S, 2050 and 1160 mAh g^−1^ with 56.6% for δ-MnO_2_-G, and 1856 and 556 mAh g^−1^ with 30.0% for δ-MnO_2_-F, respectively. The high-capacity loss at first delithiation might have originated from the irreversible formation of the solid electrolyte interface (SEI) layer with electrolyte decomposition at low voltages, which is frequently observed for metal oxide materials. Notably, a considerably lower capacity was observed in δ-MnO_2_-F. It is presumed that δ-MnO_2_-F has the highest SSA, and therefore, it provides many active sites for Li-ion storage. However, the irreversible capacity loss increases because of the formation of the SEI layer and the clogging of the small pores during the conversion reaction.

Subsequently, as listed in Table S2, the discharge/charge capacity of the second cycle showed similar values for δ-MnO_2_-S (1131 and 1044 mAh g^−1^) and δ-MnO_2_-G (1214 and 1089 mAh g^−1^), while coulombic efficiency (CE) of δ-MnO_2_-S (92.3%) was higher than δ-MnO_2_-G (89.7%), indicating the relatively low irreversibility of δ-MnO_2_-S with fast stabilization. These results confirm that the morphology, surface area, and pore structure can affect the electrochemical performance even if the phases are the same as those of δ-MnO_2_. However, further polymorph and shape control studies are necessary for improving ICE with a small irreversible capacity.

**Figure 3:**
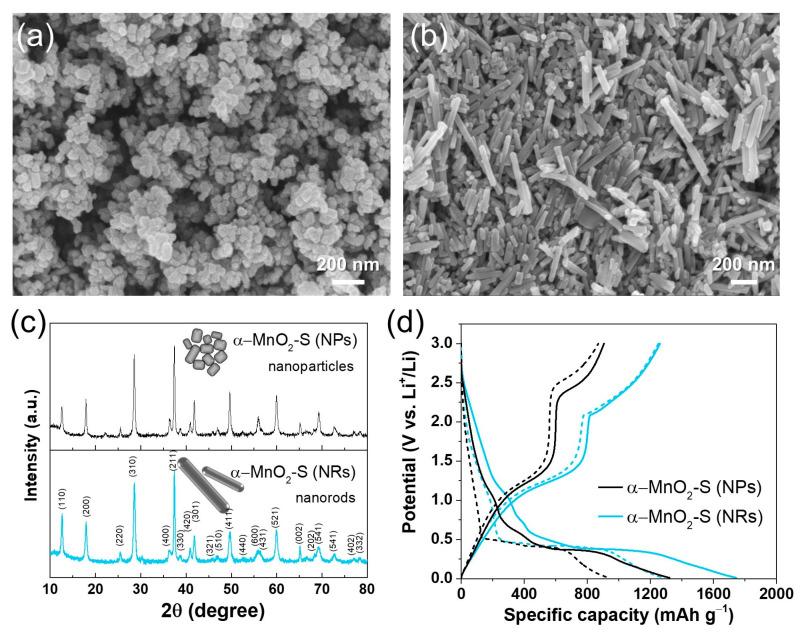
FE-SEM images of (**a**) α-MnO_2_-S-NPs (calcined at 600 °C, 5 h) and (**b**) α-MnO_2_-S-NRs (calcined at 700 °C, 2 h). (**c**) XRD patterns and (**d**) galvanostatic charge–discharge curves for two cycles of α-MnO_2_-S-NPs and α-MnO_2_-S-NRs, respectively.

α-MnO_2_ is considered a promising anode material for Li-ion batteries because of its unique tunnel structure, which facilitates the fast diffusion of Li^+^. Among the diverse morphologies, one-dimensional nanostructures have received considerable attention as promising electrode materials because of their definite advantages in terms of reaction kinetics, such as shortened ion diffusion pathways and fast axial electron transport along the one-dimensional direction [38,39]. Considering these points, we attempted to control the polymorphs and morphology using δ-MnO_2_-S because of its relatively small irreversible capacity with high ICE. Figure 3a,b show that the morphologies of the nanoparticles and nanorods are different between the two samples. The samples calcined at 600 °C for 5 h were confirmed as nanoparticles with distinct edge shapes and sub-100 nm sizes (Figure 3a); the samples calcined at 700 °C for 2 h showed a rod shape with lengths of tens to hundreds of nanometers and widths of sub-60 nm (Figure 3b). In addition, XRD patterns showed a successful polymorph transition from δ-MnO_2_ to α-MnO_2_ (JCPDS 044-0141) and an amorphous-to-crystalline transition under both calcination conditions. Therefore, they were designated by α-MnO_2_-NPs (nanoparticles) and α-MnO_2_-NRs (nanorods), respectively (Figure 3c). Furthermore, galvanostatic charge–discharge capacity tests were performed to verify the effect of morphology on electrochemical performance (Figure 3d). The specific discharge and charge capacity of the initial cycle with ICE were 1321 and 905 mAh g^−1^ with 68.5% for α-MnO_2_-NPs, and 1744 and 1260 mAh g^−1^ with 72.2% for α-MnO_2_-NRs. The α-MnO_2_-NRs shows a higher reversible capacity than that of α-MnO_2_-NPs, which suggests that morphology plays an essential role in electrochemical performance. Further, compared with δ-MnO_2_ (see Figure 2h), α-MnO_2_ exhibited higher capacity and stability. Therefore, based on these results, further studies were conducted on the morphology control and electrochemical properties of α-MnO_2_ having a one-dimensional structure, starting with δ-MnO_2_ synthesized using different types of sugars (sucrose, glucose, and fructose).

**Figure 4:**
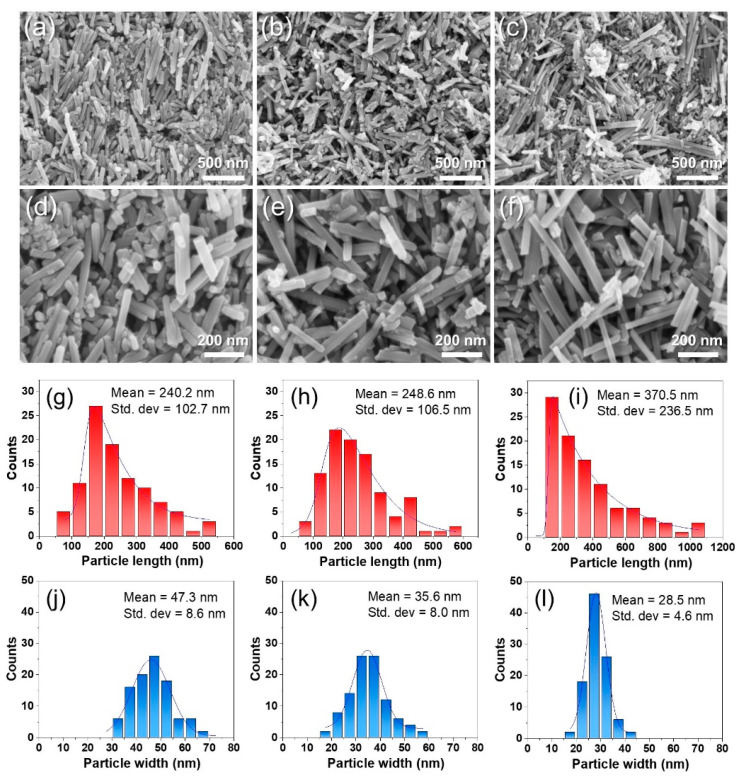
FE-SEM images taken from low- (×40 k) and high-magnification (×100 k) of (**a**,**d**) α–MnO_2_-S (**b**,**e**) α–MnO_2_-G, and (**c**,**f**) α–MnO_2_-F. Particle size distribution of length (*c*-axis) and width (*a*-axis) of (**g**,**j**) α–MnO_2_-S (**h**,**k**) α–MnO_2_-G, and (**i**,**l**) α–MnO_2_-F.

The morphologies of the obtained α-MnO_2_-S, α-MnO_2_-G, and α-MnO_2_-F were observed using FE-SEM. Figure 4a–f shows that all samples exhibited nanorod shapes with different lengths and widths. The length, width, and aspect ratio of the nanorods were measured by counting 100 particles in the FE-SEM images using ImageJ software to determine the particle size and distribution [40]. Figure 4g–i and Figure 4j–l show the length and width distribution histograms for each sample, respectively. The mean values of length were 240.2, 248.6, and 370.5 nm, and those of the widths were 47.3, 35.6, and 28.5 nm for α-MnO_2_-S, α-MnO_2_-G, and α-MnO_2_-F, respectively. The calculated aspect ratio (*c*/*a*) was 5.1, 7.0, and 13.0 for α-MnO_2_-S, α-MnO_2_-G, and α-MnO_2_-F, respectively. The different aspect ratios likely originate from the morphologies of the base materials (δ-MnO_2_). The aspect ratios are inversely proportional to the particle sizes of the base materials. The largest particle size of δ-MnO_2_-S induced the lowest aspect ratio. These morphological trends are strongly related to the base materials. We tentatively presume that the growth mechanism of the rod-shaped particles is closely related to the seed growth process via oriented attachment [41,42,43]. The aspect ratio (*c/a*) of nanorods is dependent on seed size, wherein the aspect ratio increases with small seeds and vice versa. Therefore, the structure properties, including the primary particle size and the aggregated form, of the base materials (δ-MnO_2_) can affect the particle size distribution and aspect ratio of the final products of α-MnO_2_. In addition, the SSA and pore structures of the three types of α-MnO_2_ were evaluated. All samples revealed mesoporous structure (IV isotherm model, H3 hysteresis [44]) and SSA of 20.6–21.7 m^2^ g^−1^. The pore volume and pore diameter were large, in the order α-MnO_2_-S > α-MnO_2_-G > α-MnO_2_-F, as shown in Figure S3 and Table S3. These trends are related to the nanorod shape and stacking form, which can be affected by the aspect ratio. The affection of pore structure for Li-ion pathway and transport will be discussed in the electrochemical outcome section. Additionally, to confirm the effect of sugar type on the oxidation state of Mn, peak separation (ΔE) of Mn 3s was observed by XPS analysis (Figure S4). All samples revealed almost the same value of ΔE, in which 4.76, 4.72, and 4.73 eV for α-MnO_2_-S, α-MnO_2_-G, and α-MnO_2_-F, respectively. These values are well consistent with the reported data in the literature of Mn(IV), and it is confirmed that the sugar type used as a reducing promoter did not affect the oxidation state of Mn [45].

**Figure 5:**
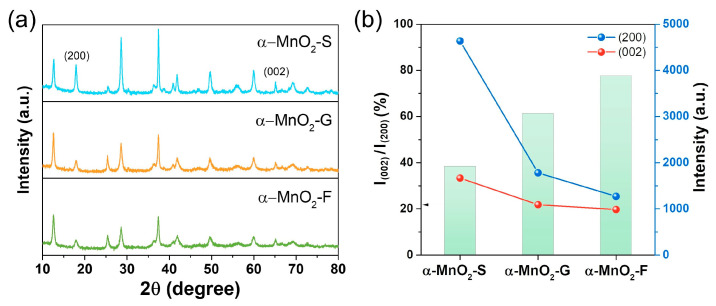
(**a**) XRD patterns of α-MnO_2_-S, α-MnO_2_-G, and α-MnO_2_-F. (**b**) Intensity ratio of (002) and (200) plane and intensities of (200) and (002) peaks of α-MnO_2_-S, α-MnO_2_-G, and α-MnO_2_-F.

The XRD patterns of all samples showed typical peaks of α-MnO_2_ (Figure 5a). However, they showed differences in relative intensity. The relative intensity ratio (*I*_(002)_/*I*_(200)_) between the (002) and (200) planes is associated with the aspect ratio (*c*/*a*) of the nanorod structure; a higher *I*_(002)_/*I*_(200)_ value implies a higher aspect ratio [46,47]. The calculated *I*_(002)_/*I*_(200)_ values were 38.4, 61.2, and 77.5% for α-MnO_2_-S, α-MnO_2_-G, and α-MnO_2_-F, respectively, which is in good agreement with the aspect ratios from the FE-SEM observations (Figure 5b). Therefore, the effects of different dimensions in each direction on the electrochemical properties were investigated.

**Figure 6:**
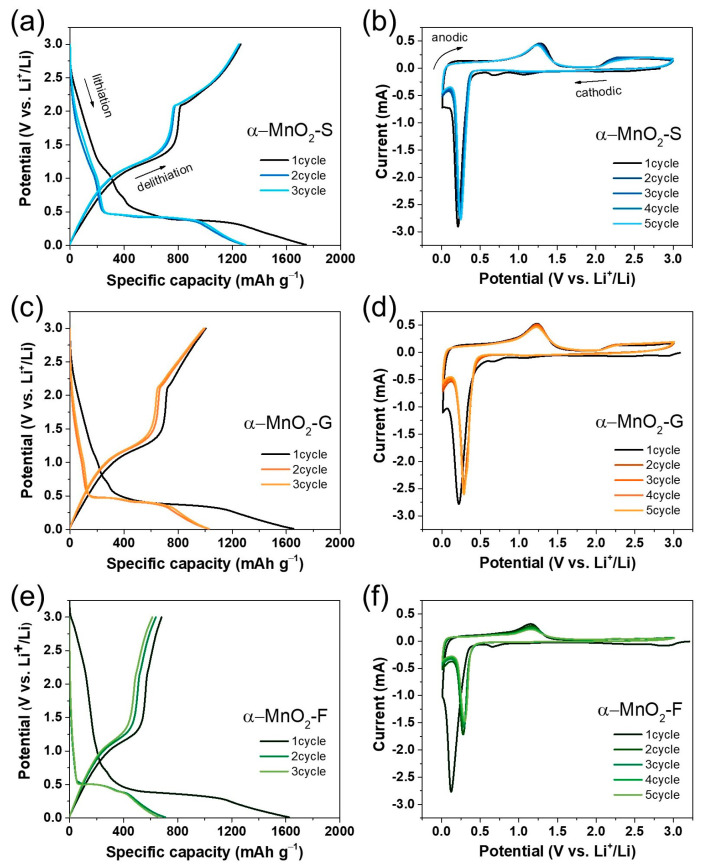
Galvanostatic discharge–charge curve for three cycles of (**a**) α–MnO_2_-S, (**c**) α–MnO_2_-G, and (**e**) α–MnO_2_-F. Cyclic voltammetry curves for five cycles of (**b**) α–MnO_2_-S, (**d**) α–MnO_2_-G, and (**f**) α–MnO_2_-F.

Figure 6a,c,e shows the galvanostatic discharge and charge voltage profiles for the initial three cycles. The first discharge curve (black line) exhibits a slope with humble peaks at around ~1.0 V and 0.6 V, indicating the formation of a solid electrolyte interface layer. Moreover, this voltage range involves the conversion of tetravalent manganese (MnO_2_) to divalent (MnO) during discharging. Following the flat plateau at around 0.38–0.4 V up to complete lithiation shows the conversion reaction of divalent MnO to metallic Mn. Conversely, the first charge plot involves two characteristic plateaus in the range of 0.1–1.6 V and 1.6–3 V, indicating reversible conversion reactions of (i) metallic Mn to divalent MnO, and (ii) divalent to tri- or tetravalent Mn [3,12,13,16]. The ICE and reversible discharge capacity were found to be 72.3% and 1287 mAh g^−1^ for α-MnO_2_-S, 60.7% and 1026 mAh g^−1^ for α-MnO_2_-G, and 41.9% and 709 mAh g^−1^ for α-MnO_2_-F. Among them, α-MnO_2_-S exhibited the highest reversible specific capacity, and CE of the second and third cycles also presented the highest values, as summarized in Table S4.

Figure 6b,d,f presents the cyclic voltammograms of α-MnO_2_ electrodes measured with a sweep rate of 0.1 mV s^−1^ for five cycles. A sharp cathodic peak at around 0.13–0.21 V and two major anodic peaks at around 0.24–0.28 V and above 2.0 V were observed in all samples. In the cathodic scan for the initial cycle, small peaks at around 0.65–0.67 V and 1.06–1.07 V were observed; meanwhile, no additional peak was observed at a similar voltage region in the following cycles, suggesting the formation and stabilization of the SEI layer during the cycle. The sharp cathodic peaks corresponding to the conversion reaction of Mn^4+^ to Mn^0^ during the first and second cycles shifted toward a high potential in the second cycle, and it was calculated to be 0.03 V (α-MnO_2_-S), 0.07 V (α-MnO_2_-G), and 0.15 V (α-MnO_2_-F). A small voltage gap indicates a small irreversible reaction during the cycles, and the smallest value of α-MnO_2_-S is consistent with the results of the ICE. In the anodic scan, two major peaks are maintained during cycles without degradation in α-MnO_2_-S and α-MnO_2_-G, whereas the peak disappears in α-MnO_2_-F, indicating poor electrochemical stability. The significant difference in the charge–discharge curve and CV profile of each sample can provide evidence regarding the effect of morphological properties on Li-ion storage.

**Figure 7:**
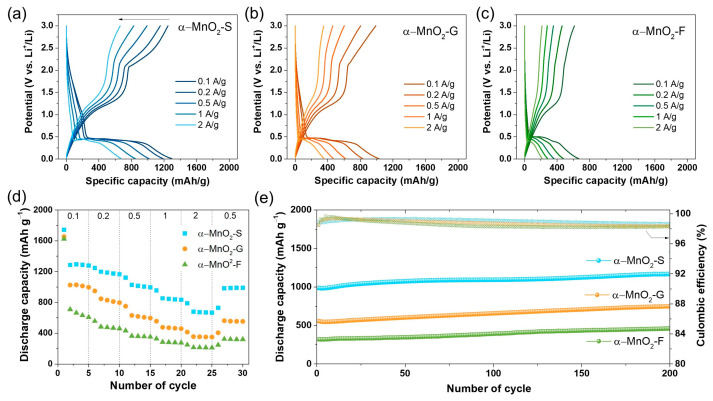
Voltage profiles at different current densities of (**a**) α-MnO_2_-S, (**b**) α-MnO_2_-G, and (**c**) α-MnO_2_-F. (**d**) Rate capability at various current densities of 0.1, 0.2, 0.5, 1, 2, and 0.5 A g^−1^. (**e**) Cycling stability and coulombic efficiency of α-MnO_2_ electrodes tested at a current density of 0.5 A g^−1^ for 200 cycles.

The rate capabilities of α-MnO_2_-S, α-MnO_2_-G, and α-MnO_2_-F were evaluated at current densities of 0.1, 0.2, 0.5, 1, 2, and 0.5 A g^−1^. Figure 7a–c shows the charge–discharge curves of the third cycle for each current density. α-MnO_2_-S exhibits a superior rate capability with the highest specific capacity increasing with the current density: 1294, 1186, 1014, 845, and 673 mAh g^−1^ at a rate of 0.1, 0.2, 0.5, 1, and 2 A g^−1^, respectively. α-MnO_2_-S delivered a reversible capacity of 987 mAh g^−1^ when the current density returned to 0.5 A g^−1^ even after 25 cycles (Figure S5); this is calculated to be 97.3%. Figure 7d shows specific discharge capacities at different current densities. α-MnO_2_-G and α-MnO_2_-F were lower than α-MnO_2_-S, and the conducted values are summarized in Table S5.

Further, we compared the cycling stability of α-MnO_2_-S, α-MnO_2_-G, and α-MnO_2_-F at a current density of 0.5 A g^−1^ for 200 cycles. Figure 7e shows the specific discharge capacity and CE with the cycle number. During cycling, all samples exhibited stable cycle retention without significant degradation. After 200 cycles, the discharge capacity was 1169, 750, and 460 mAh g^−1^ for α-MnO_2_-S, α-MnO_2_-G, and α-MnO_2_-F, respectively. In addition, the CE values were calculated to be 99.4–98.1% for 200 cycles; almost 100% of CE was attributed to the exclusion of additional SEI layer formation with repeated cycling. Moreover, the cycled electrodes did not show significant morphological changes, which presented a slightly expanded structure during conversion reactions (Figure S6). All samples showed a gradual increase in capacity with repeated cycling. Similar trends were commonly observed in conversion-type oxide-based anodes, which is attributed to the reversible growth of a pseudocapacitive polymeric gel-like film during the cycle [48,49,50,51].

**Figure 8:**
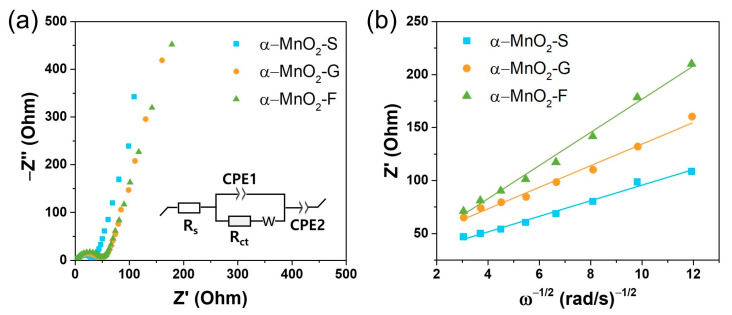
(**a**) Nyquist plots and (**b**) plots of Z′ vs. ω^−1/2^ in the low-frequency region of α-MnO_2_ electrodes.

The kinetic parameters of each electrode are determined by EIS (Figure 8). The obtained Nyquist plots are fitted using the equivalent circuit model shown in Figure 8a; the kinetic parameters are listed in Table S6. The determined charge transfer resistances (*R*_ct_) are 32.5, 46.3, and 49.5 Ω for α-MnO_2_-S, α-MnO_2_-G, and α-MnO_2_-F, respectively. The obtained *R*_ct_ tends to increase with an increasing aspect ratio (*c*/*a*) of the nanorods. Furthermore, the Warburg coefficient (σ), which is associated with the tail in the low-frequency region, is used to evaluate the effect of the morphologies. σ represents the slope of the real impedance versus the angular frequency, and a small σ value implies higher ion diffusivity in the interior of the particles [52,53]. The slope gradually increases in the order of α-MnO_2_-S, α-MnO_2_-G, and α-MnO_2_-F, exhibiting the same trend as that for *R*_ct_ (Figure 8b). The EIS results suggested a morphological difference, i.e., a small aspect ratio (*c*/*a*), resulting in a reduced *R*_ct_ and fast Li-ion diffusion at the electrode/electrolyte interface and interior. For these reasons, the α-MnO_2_-S electrode presented the highest capacity and rate capability.

**Figure 9:**
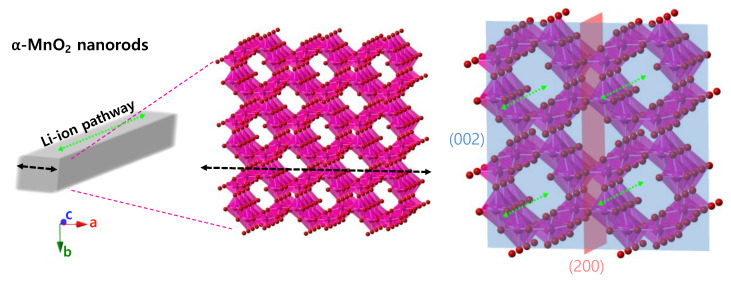
Schematic of the correlation between the nanorod’s shape and crystallographic structures on the Li-ion transport mechanism.

The mechanism of the correlation between the nanorod shape and its crystallographic structure is schematically shown in Figure 9. All nanorod shapes of α-MnO_2_ grown from each δ-MnO_2_ were elongated along the *c*-axis. The lengths and widths of nanorods represent the *ab*-plane and *bc*-plane, respectively. The FE-SEM observations confirmed that α-MnO_2_-S revealed the largest width and shortest length among the prepared nanorods. The increase in width (i.e., the grown *bc*-plane) implies an increase in the tunnel structure. In fact, the Li ions migrate through the tunnel along the *c*-axis [54,55]. Consequently, the largest width of α-MnO_2_-S provides an increased number of pathways for Li ions. Therefore, an increased Li-ion pathway contributes to sufficient ion transport, whereas a relatively shorter *ab*-plane results in a shorter diffusion length. These crystallographic features mainly contributed to achieving a higher rate capability with a high capacity. In addition, their larger pore volume and size compared to those of other electrodes provide high accessibility to the electrolyte, leading to an increase in the electrode/electrolyte interface for fast ion transport.

On the basis of the above results, α-MnO_2_-S exhibited the best battery performance because of its controlled polymorphism, shape, and aspect ratio. These findings reveal the morphological effects on electrochemical properties and strategies for controlling the morphologies of materials. In particular, a determination of the optimal aspect ratio and size effect on Li-ion transportation will provide useful information for the rational design of manganese-based anode materials. Further, the α-MnO_2_-S electrode without any composite conductive carbon-based materials achieved superior performance with a high-rate capability compared to the literature on MnO_x_ and/or carbon composite anodes (Table S7). Thus, the construction of α-MnO_2_-S/carbon composites, such as graphene or carbon nanotubes, further improved their performance in future studies.

## 4. Conclusions

Birnessite δ-MnO_2_ nanoparticles were synthesized using SPP with three types of sugar. The as-synthesized δ-MnO_2_ exhibited different particle sizes and SSA owing to the different nucleation rates and reaction times originating from the inherent molecular structure of the sugars. The relatively larger particles (~200 nm) with smaller SSA of δ-MnO_2_-S exhibited the best ICE and rate capability among the three types of δ-MnO_2_. Subsequently, the polymorph transformed to α-MnO_2_ showed improved battery performance in the form of nanorods compared to that for nanoparticles. In addition, the three types of α-MnO_2_ nanorods revealed different aspect ratios (*c/a*), which were highly related to the morphologies of the based δ-MnO_2_. The FE-SEM and XRD analysis confirmed that the larger particle size of the based δ-MnO_2_ led to varied widths of nanorods formed with a seed growth process via oriented attachment. A relatively small aspect ratio (*c/a* = 5.1) with an expanded width could provide sufficient Li-ion transport in the interior of the electrode by increasing the ion pathway, whereas a larger pore volume and size contributed to the fast Li-ion transport at the electrode/electrolyte interface. Thus, α-MnO_2_-S exhibited an excellent rate performance of 673.4 mAh g^−1^ at 2 A g^−1^. We expect these strategies for polymorphism and aspect ratio control to be promising for designing advanced anode materials as well as other energy storage materials.

## Data Availability

The data that support the findings of this study can become available by the corresponding authors upon reasonable request.

## Supplementary Materials

The following supporting information can be downloaded at: https://www.mdpi.com/article/10.3390/nano13202808/s1, Figure S1: Experimental setup of the solution plasma process (SPP); Figure S2: (a) Specific surface area and (b) pore size distribution of δ-MnO_2_ particles; Figure S3: (a) Specific surface area and (b) pore size distribution of α-MnO_2_ particles; Figure S4: XPS spectrum of Mn 3s for α-MnO_2_-S, α-MnO_2_-G, and α-MnO_2_-F; Figure S5: Comparison of galvanostatic discharge-charge curves for initial (solid line) and after rate test (dashed line) at the current density of 0.5 A g^−1^ of (a) α-MnO_2_-S, (b) α-MnO_2_-G and (c) α-MnO_2_-F; Figure S6: FE-SEM images of cycled electrodes for (a) α-MnO_2_-S, (b) α-MnO_2_-G and (c) α-MnO_2_-F.; Table S1: Summary of SSA, pore size and volume of δ-MnO_2_ particles; Table S2: Galvanostatic discharge/charge capacity for initial and second cycle for δ-MnO_2_ electrodes; Table S3: Summary of SSA, pore size and volume of α-MnO_2_ particles; Table S4: Galvanostatic discharge/charge capacity for initial, second and third cycle for α-MnO_2_ electrodes; Table S5: Specific discharge capacity in third cycle of α-MnO_2_ electrodes at various current density; Table S6: Summary of impedance parameters: Ohmic (R*_s_*), charge transfer (R*_ct_*) and Warburg coefficient (σ) of α-MnO_2_ electrodes; Table S7: Summary of cyclability and rate performance of MnO_x_ and carbon composites anodes for LIBs.
