# Supplementary material for: High-Rate One-Dimensional α-MnO2 Anode for Lithium-Ion Batteries: Impact of Polymorphic and Crystallographic Features on Lithium Storage
Source: Nanomaterials (Basel). 2023 Oct 23;13(20):2808. doi: 10.3390/nano13202808 (PMC10609827; doi:10.3390/nano13202808)
Supplement: Supplementary file 1 [file nanomaterials-13-02808-s001.zip › nanomaterials-2679970-supplementary.pdf]

Supplementary Materials

High-Rate One-Dimensional α-MnO_2 Anode for Lithium-Ion Batteries: Impact of Polymorphic and Crystallographic Features on Lithium Storage

Hye-min Kim [1], Byung-chul Cha [2,*] and Dae-wook Kim [2,*]

[1] Department of Materials Chemistry, Shinshu University, 4-17-1, Wakasato, Nagano 3808553, Japan; hmkim545@gmail.com
[2] Advanced Manufacturing Process R&D Group, Ulsan Division, Korea Institute of Industrial Technology (KITECH), 55, Jongga-ro, Jung-gu, Ulsan 44313, Republic of Korea
* Correspondence: bccha76@kitech.re.kr (B.-c.C.); dwkim@kitech.re.kr (D.-w.K.)

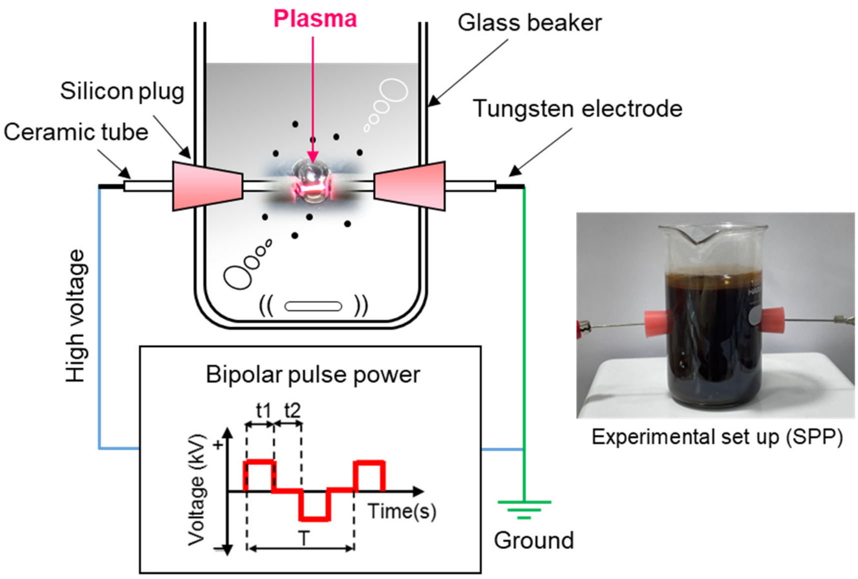

Figure S1 Experimental set-up of solution plasma process (SPP)

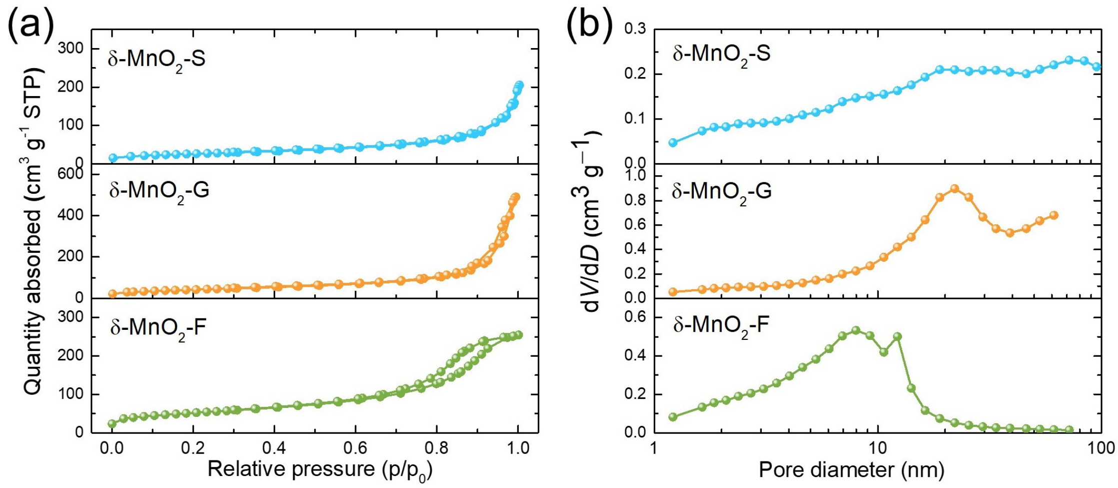

Figure S2 (a) Specific surface area and (b) pore size distribution of δ-MnO_2 particles.

Table S1 Summary of SSA, pore size and volume of δ-MnO_2 particles.

Sample	Specific surface area (m^2 g^{-1})	Pore volume (cm^3 g^{-1})	Avg. pore size (nm)
δ-MnO_2-S	83.8	0.31	14.4
δ-MnO_2-G	139.4	0.75	20.9
δ-MnO_2-F	165.9	0.38	9.0

Table S2. Galvanostatic discharge/charge capacity for initial and second cycle for δ-MnO_2 electrodes.

		Specific discharge capacity (mAh g^{-1})		
		δ-MnO_2-S	δ-MnO_2-G	δ-MnO_2-F
Initial cycle	Dch.	1689	2050	1856
	Char.	1090	1160	556
	I.C.E (%)	64.5	56.6	30.0
Second cycle	Dch.	1131	1214	611
	Char.	1044	1089	517
	C.E (%)	92.3	89.7	84.6

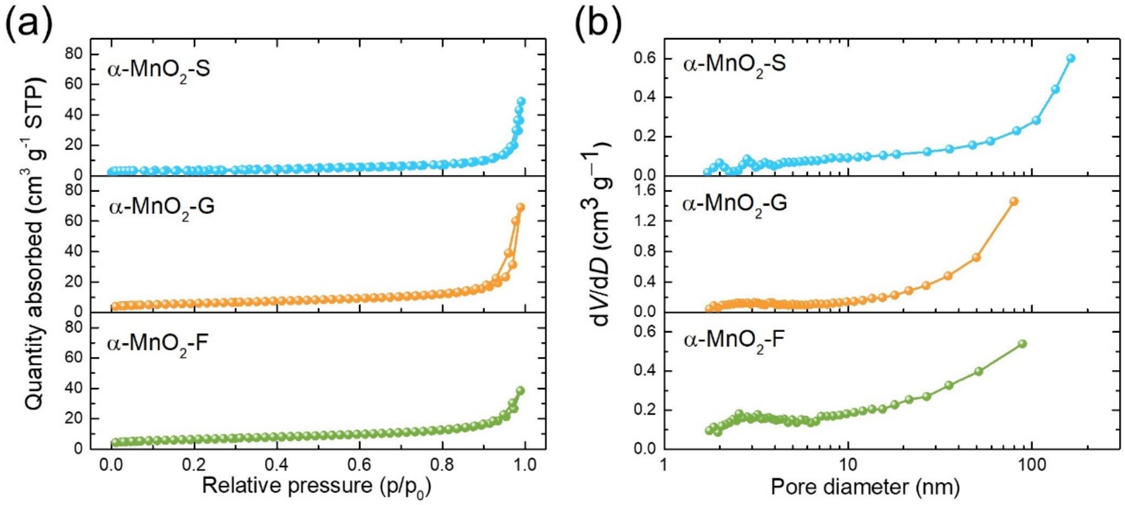

Figure S3 (a) Specific surface area and (b) pore size distribution of α-MnO_2 particles.

Table S3 Summary of SSA, pore size and volume of α-MnO_2 particles.

Sample	Specific surface area (m^2 g^{-1})	Pore volume (cm^3 g^{-1})	Avg. pore size (nm)
α-MnO_2-S	21.3	0.18	33.1
α-MnO_2-G	20.6	0.11	20.8
α-MnO_2-F	21.7	0.06	10.9

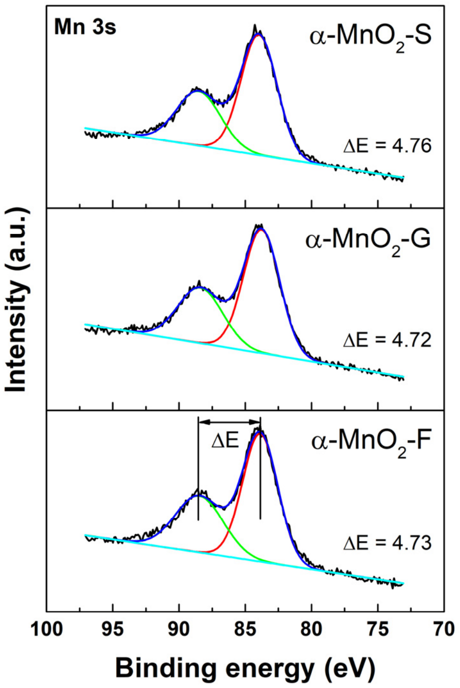

Figure S4 XPS spectrum of Mn 3s for α-MnO_2-S, α-MnO_2-G and α-MnO_2-F

Table S4. Galvanostatic discharge/charge capacity for initial, second and third cycle for α-MnO_2 electrodes.

		Specific discharge capacity (mAh g^{-1})		
		α-MnO_2-S	α-MnO_2-G	α-MnO_2-F
Initial cycle	Dch.	1744	1654	1626
	Char.	1260	1004	680
	I.C.E (%)	72.2	60.7	41.8
Second cycle	Dch.	1287	1026	709
	Char.	1251	997	638
	C.E (%)	97.2	97.2	90.0
Third cycle	Dch.	1294	1027	664
	Char.	1248	989	612
	C.E (%)	96.4	96.3	92.2

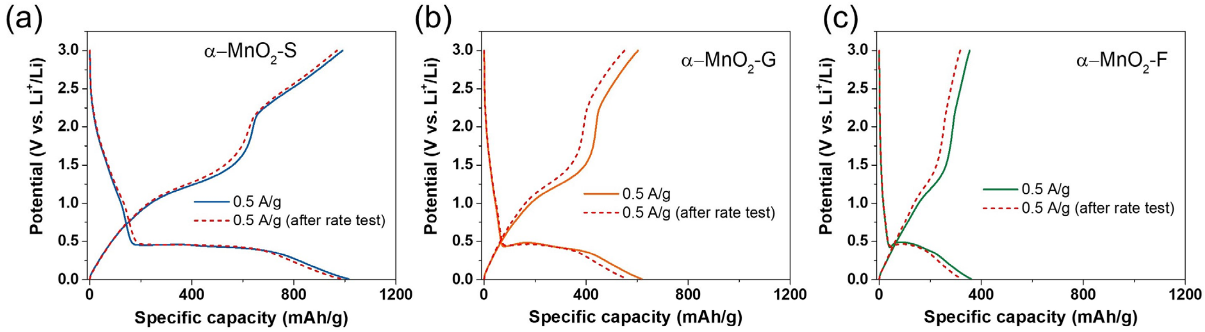

Figure S5 Comparison of galvanostatic discharge-charge curves for initial (solid line) and after rate test (dashed line) at current density of 0.5 A g^{-1} of (a) α-MnO_2-S, (b) α-MnO_2-G and (c) α-MnO_2-F.

Table S5 Specific discharge capacity in third cycle of α-MnO_2 electrodes at various current density.

Current Density (A g^{-1})	Specific discharge capacity (mAh g^{-1})			Rate capability % (relative retention) (Rate/0.1 C)		
	α-MnO_2-S	α-MnO_2-G	α-MnO_2-F	α-MnO_2-S	α-MnO_2-G	α-MnO_2-F
0.1	1294	1027	664	100	100	100
0.2	1186	828	476	91.7	80.6	71.7
0.5	1014	618	361	78.4	60.2	54.4
1	845	470	283	65.3	45.8	42.6
2	673	353	215	52.0	34.4	32.4
0.5	987	556	321	76.3	54.1	48.3

Table S6. Summary of impedance parameters: Ohmic (R_s), charge transfer (R_{ct}) and Warburg coefficient (σ) of α-MnO_2 electrodes.

	R_s (ohm)	R_{ct} (ohm)	σ ($\Omega\ s^{-1/2}$)
α-MnO_2-S	1.8	32.5	7.3
α-MnO_2-G	2.0	46.3	10.2
α-MnO_2-F	1.6	49.5	15.8

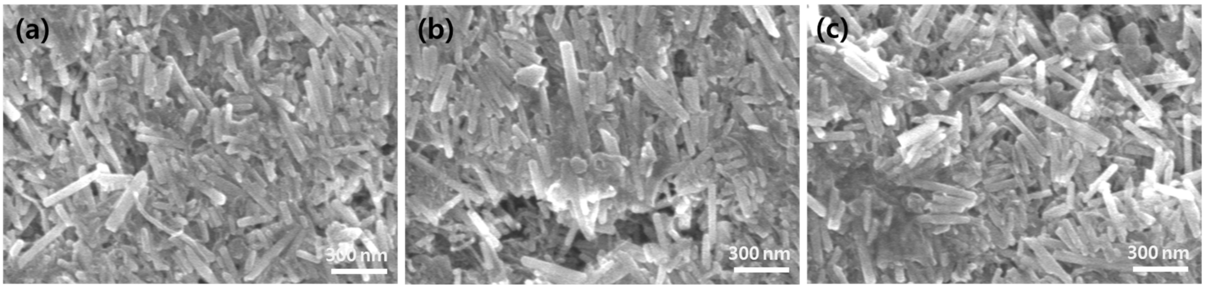

Figure S6 FE-SEM images of cycled electrodes for (a) α-MnO_2-S, (b) α-MnO_2-G and (c) α-MnO_2-F.

Table S7. Summary of cyclability and rate performance of MnO_x and carbon composites anodes for LIBs

Electrode materials	Rate performance (mAh g^{-1})	Ref.
**MnO_2/3D-rGO**	380 (1 A/g)	Ref 1
**δ-MnO_2 nanoscrolls/RGO**	226 (1A/g)	Ref 2
**Nanostructured MnO_2**	464 (2 A/g)	Ref 3
**MnO_2–3D Porous**	433 (1.6 A/g)	Ref 4
**MCM-MnO_2**	65 (2.4 A/g)	Ref 5
**MnO_2/VACNTs (vertically aligned carbon nanotubes)**	414 (2 A/g)	Ref 6
**$NiCo_2O_4$@MnO_2**	398 (1.6 A/g)	Ref 7
**MnO_2@HCN (Hollow carbon nanosphere)**	150 (2 A/g)	Ref 8
**Mn_2O_3**	528.4 (2 A/g)	Ref 9
**α-MnO_2/graphene**	340 (2 A/g)	Ref 10
**MnO_x@C**	670 (2A/g)	Ref 11
**δ-MnO_2/C**	212 (1.5 A/g)	Ref 12
**SnO_2@MnO_2@graphite nanosheet**	621.6 (2 A/g)	Ref 13
**MnO_2-polypyrrole nanorods**	475 (2 A/g)	Ref 14
**MnO_2 nanosheets**	576 (2 A/g)	Ref 15
**Fe_3O_4@C@MnO_2**	445 (2 A/g)	Ref 16
**Mn_3O_4/NHC (N-doped honeycomb carbon)**	472 (2 A/g)	Ref 17
**MnO_2 nanorods**	498.2 (1 A/g)	Ref 18
**MnO_2@Fe_2O_3 composite**	583.1 (1.6 A/g)	Ref 19
**α-MnO_2-S**	**673 (2 A/g)**	This work
**α-MnO_2-G**	**353 (2 A/g)**	This work
**α-MnO_2-F**	**215 (2 A/g)**	This work

Reference

[1] H. Liu, Z. Hu, Y. Su, H. Ruan, R. Hu, L. Zhang, Appl. Surf. Sci., 2017, 392, 777-784.

[2] H. Liu, Z. Hu, L. Tian, Y. Su, H. Ruan, L. Zhang, R. Hu, Ceram. Int., 2016, 42, 13519-13524.

[3] L. Zhang, J. Song, Y. Liu, X. Yuan, S. Guo, J. Power Sources, 2018, 379, 68-73.

[4] Y. Li, Q. Zhang, J. Zhu, X.-L. Wei, P.K. Shen, J. Mater. Chem. A, 2014, 2, 3163-3168.

[5] P.M. Ette, K. Selvakumar, S.M.S. Kumar, K. Ramesha, Electrochim. Acta, 2018, 292, 532-539.

[6] A. Abdollahi, A. Abnavi, F. Ghasemi, S. Ghasemi, Z. Sanaee, S. Mohajerzadeh, Electrochim. Acta, 2021, 390, 138826.

[7] Z. Zhang, Y. Huang, J. Yan, C. Li, X. Chen and Y. Zhu, Appl. Surf. Sci., 2019, 473, 266-274.

[8] Z. Cao, Y. Yang, J. Qin and Z. Su, J. Power Sources, 2021, 491, 229577.

[9] S. Shi, S. Deng, M. Zhang, M. Zhao, G. Yang, Electrochim. Acta, 2017, 224, 285-294.

[10] H. S. Kim, D. W. Kim, S.-S. Kim, C. Senthil and H. Y. Jung, Chem. Eng. J., 2022, 427, 130937.

[11] Y. Ye, C. Zhu, X. Guo and F. Cheng, Energy & Fuels, 2022, 36, 5989-5997.

[12] Y. Lin, H. Tian, J. Qian, M. Yu, T. Hu, U. Lassi, Z. Chen and Z. Wu, Materials Today Chemistry, 2022, 26, 101023.

[13] W. Li, X. Deng, Y. Feng, D. Xiong and M. He, Ceram. Int., 2021, 47, 33405-33412.

[14] M. Wang, Q. Yang, T. Zhang, B. Zhu, G. Li, FRSC Adv., 2016, 6, 19952-19956.

[15] J. Zang, J. Ye, H. Qian, Y. Lin, X. Zhang, M. Zheng, Q. Dong, Electrochim. Acta, 2018, 260, 783-788.

[16] Y. Wang, S. Rao, P. Mao, F. Zhang, P. Xiao, L. Peng and Q. Zhu, Electrochim. Acta, 2020, 337, 135739.

[17] D. Zhang, C. Zhang, X. Zheng, Y. Zhao, X. Shi, B. Luo, Y. Li, G. Liu, X. Liu and C. Yu, International Journal of Minerals, Metallurgy and Materials, 2023, 30, 1152-1161.

[18] J. Chen, Y. Wang, X. He, S. Xu, M. Fang, X. Zhao, Y. Shang, Electrochim. Acta, 2014, 142, 152-156.

[19] C. Liu, X. Zhang, H. Shang, S. Li, Z. Wen, S. Ji and J. Sun, J. Alloys Compd., 2021, 876, 160113.
